# Synergistic Post-Transcriptional Regulation of the *Cystic Fibrosis Transmembrane conductance Regulator* (*CFTR*) by miR-101 and miR-494 Specific Binding

**DOI:** 10.1371/journal.pone.0026601

**Published:** 2011-10-20

**Authors:** Francesca Megiorni, Samantha Cialfi, Carlo Dominici, Serena Quattrucci, Antonio Pizzuti

**Affiliations:** 1 Department of Experimental Medicine, Sapienza University of Rome, Rome, Italy; 2 Department of Pediatrics and Infantile Neuropsychiatry, Sapienza University of Rome, Rome, Italy; Stanford University School of Medicine, United States of America

## Abstract

microRNAs (miRNAs) are a class of regulatory small non-coding molecules that control gene expression at post-transcriptional level. Deregulation of miRNA functions affects a variety of biological processes also involved in the etiology of several human mendelian and complex diseases. Recently, aberrant miRNA expression has been observed in Cystic Fibrosis (CF), an autosomal-recessive genetic disorder caused by mutations in the *CFTR* gene, in which a genotype-phenotype correlation is not always found. In order to determine miRNA role in *CFTR* post-transcriptional regulation, we searched for miR-responsive elements in the *CFTR* 3′-UTR. *In silico* analysis, performed using different computational on-line programs, identified some putative miRNAs. Both miR-101 and miR-494 synthetic mimics significantly inhibited the expression of a reporter construct containing the 3′-UTR of *CFTR* in luciferase assays. Interestingly, miR-101/miR-494 combination was able to markedly suppress *CFTR* activity by approximately 80% (p<0.001). This is one of the first *in vitro* studies implicating microRNAs as negative regulators of the *CFTR* gene expression. miRNA aberrant expression and function might explain the wide phenotypic variability observed among CF patients.

## Introduction

Cystic Fibrosis (CF) is a common monogenic disease caused by mutations in the *Cystic Fibrosis Transmembrane conductance Regulator* (*CFTR*) gene coding for an ATP-dependent anion channel-protein required to regulate sweat, digestive juices and mucus components [1]. Even if CF is inherited as a recessive trait, with more than 1500 causative mutations, a wide range of inter- and intra-familial variability is reported so that individuals with the same genotype may show different phenotypes. Therefore, the clinical phenotype is not entirely due to the *CFTR* mutant homozygous or compound heterozygous status but genetic modifiers and environmental factors are likely to modulate the severity of the disease [2], [3]. Recent papers focused on the possible role of microRNAs in the inter- and intra-familial variability of the CF clinical manifestations [4]. MicroRNAs are evolutionarily conserved, small non-coding RNAs that negatively regulate gene expression at the post-transcriptional level by either repressing translation or decreasing mRNA stability [5], [6]. Mature miRNA, a single-stranded RNA with a length of about 22 nucleotides, is incorporated into a RNA-induced silencing complex (RISC) and is able to bind target transcripts through base pairing with their 3′-untranslated regions (UTRs). The main sequence for mRNA recognition encompasses bases 2 to 8 at the 5′-end of the mature miRNA and it is known as the “seed sequence” [7], [8]. To date, more than 800 miRNAs have been computationally identified in the human genome [9], [10], each one having the potential capacity to bind to hundreds of transcripts, and the biological functions of most miRNAs are rapidly emerging. As estimated by *in silico* prediction algorithms [10], [11], miRNAs regulate at least 30% of the protein-encoding genes and are involved in a broad range of cellular processes such as proliferation, differentiation, homeostasis and apoptosis. It is therefore predictable that the dysregulation of the miRNA pathway might contribute to human diseases, including mendelian disorders as demonstrated for Atassia telangectasia, Fragile X syndrome and Huntington's disease [12]. Emerging evidence suggests that changes in expression of microRNAs are associated with Cystic Fibrosis [4], [13]–[15]. In a recent work, Oglesby et al. [13] performed a miRNA profile screening in bronchial brushings from CF individuals versus non-CF controls and showed that miR-126 was specifically down-regulated in CF airway epithelial cells and targeted *TOM1* gene transcripts. In the present work, we address the role of miRNAs in the post-transcriptional regulation of the *CFTR* gene. In particular, after the *in silico* identification of a list of putative miRNAs able to target *CFTR* mRNA, we demonstrated by *in vitro* analysis that miR-101 and miR-494 were able to markedly suppress *CFTR* expression either alone and in combination.

## Materials and Methods

### Bioinformatic analysis

UCSC (http://genome.ucsc.edu/, hg19 assembly), NCBI (http://www.ncbi.nlm.nih.gov/) and UTRdb (http://utrdb.ba.itb.cnr.it/) genome browsers provided information of human *CFTR* gene (NM_000492.3; 3HSAR032708). Computational identification of putative miRNAs targeting *CFTR* 3′-UTR was performed by the common prediction algorithms TargetScan (http://www.targetscan.org, release 5.1), PicTar (http://pictar.mdc-berlin.de/), miRBase (http://microrna.sanger.ac.uk, release 13.0), miRanda (http://www.microrna.org), EIMMo (http://www.mirz.unibas.ch/ElMMo2/) and miRDB (http://mirdb.org/miRDB/). For most programs, standard parameter settings were used. Seeds were usually considered of 6–8 bases in length, beginning at position 2 at 5′-end of the microRNA, with no mismatches or loops allowed; a single G:U wobble was acceptable only in 7- or 8-mers. We also used the meta-server miRecords (http://mirecords.biolead.org/) that integrates the predicted targets of the following miRNA target prediction tools: DIANA-microT, MicroInspector, miRanda, MirTarget2, miTarget, NBmiRTar, PicTar, PITA, RNA22, RNAhybrid, and TargetScan/TargertScanS [16]. Secondary structure and mean free energy (mfe) of the miRNA:mRNA hybrid were obtained by RNAHybrid (http://bibiserv.techfak.uni-bielefeld.de/rnahybrid/) and rna22 (http://cbcsrv.watson.ibm.com/rna22.html web servers. Furthermore, miRNA information and expression profiles, such as quantities and tissue specificity from microarray and quantitative real-time polymerase chain reaction experiments, were obtained by miRBase (http://www.mirbase.org/), miRNAMap 2.0 (http://mirnamap.mbc.nctu.edu.tw/), and miRanda [17] database interrogation.

### Luciferase reporter plasmid construction

The 3′-UTR region of CFTR (813–1553 bp of 3HSAR032708) containing the predicted target sites of miR-101 and miR-494 was amplified from human genomic DNA using a proof reading Phusion High-Fidelity PCR master mix (Finnzymes, Espoo, Finland) with the following primers CFTR 3′UTR-F 5′-GCTCTAGAAGACCTTTGAACTAGAGTTTAGC-3′ and CFTR 3′UTR-R 5′-GCTCTAGAACACAAATGTATGGATTTTATTG -3′. The amplified 741-bp product was inserted into the XbaI site (underlined primer sequences) of the pRLTK vector (Promega) immediately downstream of the luciferase gene. Transformants were validated by specific restriction digestions and direct sequencing. The luciferase reporter construct was termed pCFTR-3′UTR. The CFTR luciferase vectors with mutated target sites for miR-101 or miR-494 (pCFTR-3′UTR-mut101 and pCFTR-3′UTR-mut494, respectively) were generated using the Quickchange II Site-directed mutagenesis kit (Stratagene, Foster City, CA) and synthetic oligonucleotides (Sigma-Aldrich):

mut101-F 5′-CTGACTCTTAAGAAGACTGCATTATATTTAT**TAGAGAT**AGAAAATATCACTTGTC-3′ and mut101-R 5′-GACAAGTGATATTTTCT**ATCTCTA**ATAAATATAATGCAGTCTTCTTAAGAGTCAG-3′; mut494-F 5′-CTCTAGGAAATATTTATTTTAATA**AACTTACG**AACATATATAACAATGCTG-3′ and mut494-R 5′-CAGCATTGTTATATATGTT**CGTAAGTT**TATTAAAATAAATATTTCCTAGAG-3′.

### Cell culture

Human embryonic kidney 293 (HEK293) cells, purchased from ATCC (Manassas, VA), were cultured in DMEM (Gibco, Carlsbad, CA) supplemented with 10% fetal bovine serum, 1% non-essential aminoacid solution, 1% L-glutamine and 2% penicillin/streptomycin. Cells were incubated at 37°C and 5% CO_2_.

### Transfection of microRNAs and reporter plasmids for luciferase assays

HEK293 were plated at a density of 3×10^5^ per well in 24-well plates and transiently transfected after 4–5 hours with 50 ng of Renilla luciferase expression constructs (pRLTK, pCFTR-3’UTR, pCFTR-3’UTR-mut101 or pCFTR-3′UTR-mut494), 12.5 ng of reference Firefly luciferase reporter (pGL3-SV40, Promega) and miRNA duplexes at 100 nM final concentration (hsa-mir-101 miRIDIAN Mimics MI0000739/MIMAT0000099, hsa-mir-494 miRIDIAN MimicsMI0003134/MIMAT0002816, miRIDIAN microRNA Mimic Negative Control #1, Dharmacon, Inc. Chicago, IL) using 1.5 µl of Lipofectamine 2000 (Invitrogen) in OptiMEM reduced serum media (Invitrogen) in accordance with the recommended conditions. Cells were lysed 48 h after transfection and luciferase assays were performed using the Dual Luciferase Reporter Assay System (Promega) following the manufacturer′s protocol. Renilla luciferase activity was normalized to the Firefly luciferase activity for each reaction. The experiments were carried out at least three times, each in triplicate. All the luciferase data were expressed as the mean ± standard error (SE) normalized to the negative control miRNA for the same reporter construct.

### Statistical analysis

Statistical significance of the comparison between two or multiple treatments was derived from one-way analysis of variance (ANOVA) for independent samples with Bonferroni post-test using GraphPad software. Differences were considered significant if the probability (p) was <0.05. The number of asterisks indicates the p-value (*, p<0.05; **, p<0.01; *** p<0.001).

## Results

**Figure 1:**
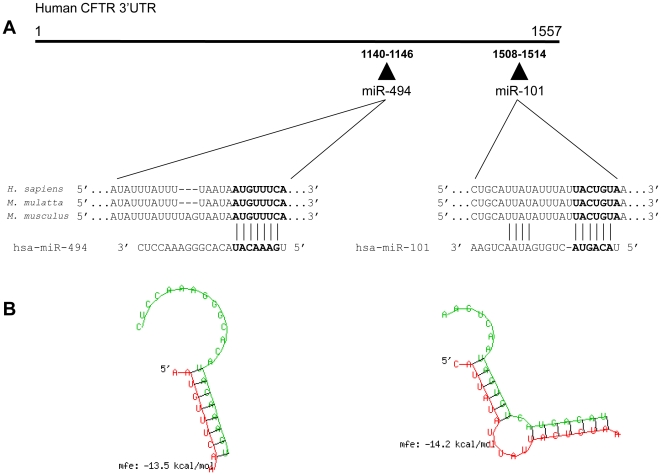
Bioinformatic prediction of microRNAs potentially targeting the *CFTR* gene. (A) Schematic representation of the *CFTR* 3’UTR and miRNAs (triangles). Ribonucleotide sequences of the putative miR-101 and miR-494 responsive elements in aligned human, rhesus and mouse *CFTR* 3’UTRs paired with the mature human miR-sequence (from TargetScan 5.1 database). Numbers indicate the predicted miR-101 and miR-494 seed sequences (in bold) using the numbering of the human *CFTR* 3’UTR (3HSAR032708 from UTRdb database). (B) Predicted *CFTR* 3’UTR hybrid structure with miR-101 or miR-494 and mean free energy (mfe) obtained by RNAHybrid server.

Computational prediction of microRNA responsive elements (MREs) within the 3′-UTR of *CFTR* transcript was performed using commonly prediction programs such as TargetScan, PicTar, and miRanda. These web servers use complicated algorithms searching for target sequences with perfect or nearly perfect pairing to the 3′-UTR sequence, evaluating the thermodynamic stability of miRNA-mRNA hybrids and performing comparative sequence analysis to check evolutionary conservation. Also, the 3′ pairing contribution, the local AU content and the distance to the nearest end of the annotated UTR were scored. Since various programs might predict different microRNAs for a given coding gene, we also used the “Predicted Targets” section of miRecords which gave a global view of our analyses as this meta-server integrates predicted miRNA targets produced by 11 established miRNA target prediction programs. Overall, we registered 496 putative *CFTR* targeting miRNAs. Three hits were predicted by five single programs and 20 hits by four target prediction softwares. The most likely candidate miRNAs targeting the *CFTR* 3’UTR which overlapped in at least three prediction programs resulted miR-101, miR-144, miR-199-3p, miR-345, miR-376b, miR-377, miR-380, miR-494, miR-509-3p, miR-600 and miR-645. We combined all these information with thermodynamic and on-line available expression data and, finally, we selected miR-101 and miR-494 microRNAs as the more likely regulators of the *CFTR* mRNA. Notably, these miRNAs looked up-regulated in the miRNA expression profile analysis performed in the CF bronchial brushings in comparison to healthy controls [13]. Both miR-101 and miR-494 are conserved in *Homo sapiens*, *Macaca mulatta* and *Mus musculus* and each miRNA sequence has a single putative target site within the *CFTR* 3’UTR at position 1508–1514 and 1140–1147, respectively (Figure 1A). In particular, miR-101 responsive element is a 7mer-A1 having an exact match to positions 2–7 of the miRNA (the seed) and an immediate downstream 'A' which is across from microRNA nucleotide 1 while miR-494 target site is a 8-mer element that shares an exact complementarity to position 2–8 of the mature microRNA followed by an 'A'. The miR-101:*CFTR* mRNA heteroduplex displayed a mfe value of −14.2 kcal/mol whereas miR-494:*CFTR* hybrid structure showed a −13.5 kcal/mol value (Figure 1B).

**Figure 2:**
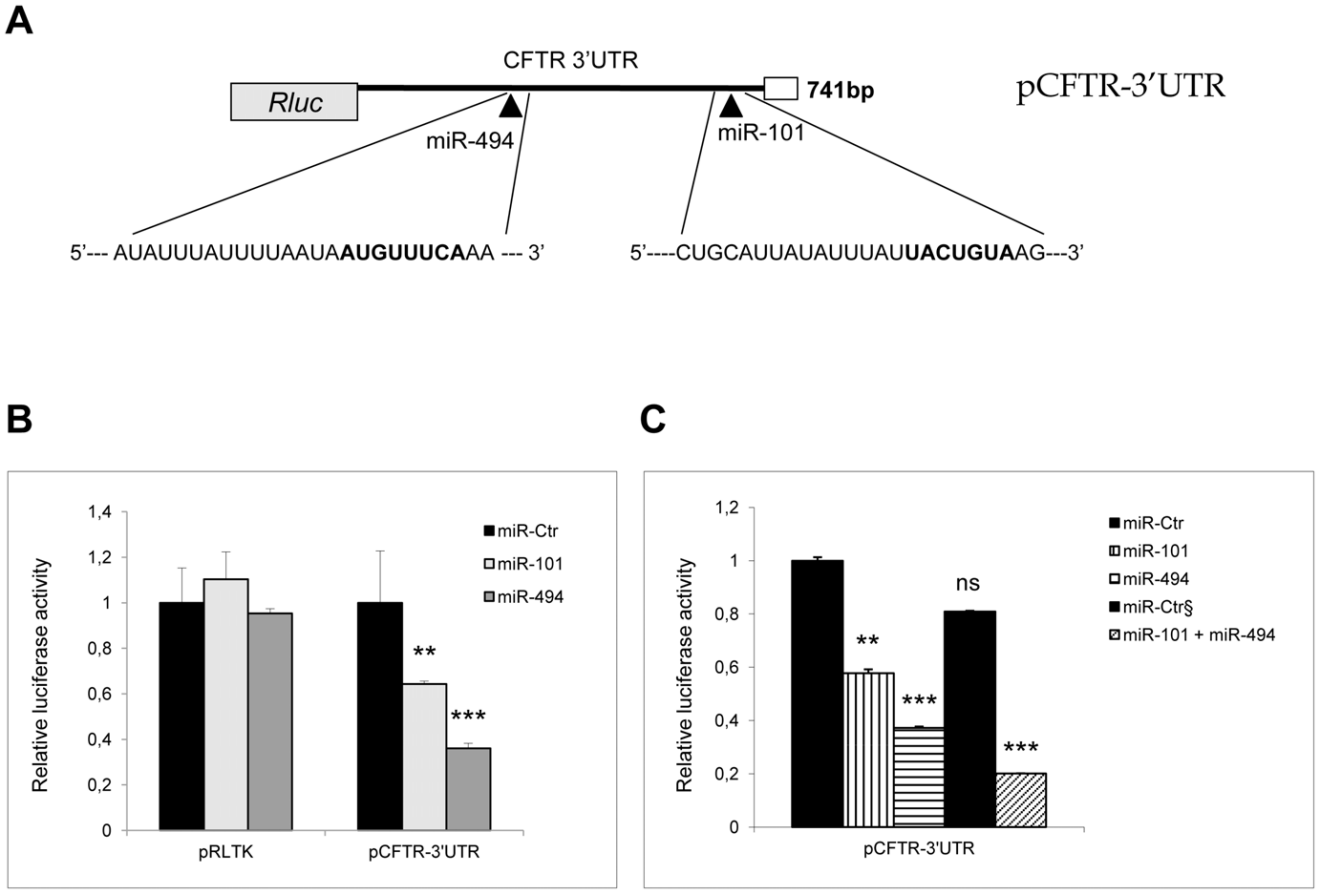
Mir-101 and mir-494 target the *CFTR* 3’-UTR. (A) Schematic representation of the construct used in the luciferase assays. A fragment of 741 bp of the *CFTR* 3’-UTR, encompassing the putative responsive elements for miR-101 and miR-494, was cloned in pRLTK vector downstream to the Renilla luciferase coding sequence. (B) HEK293 cells were transfected independently with pRLTK control plasmid or pCFTR-3’UTR vector together with either specific microRNA (miR-101 or miR-494) or a negative control miRNA (miR-Ctr). At 48 hour post-transfection, luciferase activity was measured and normalized to the Firefly control. Data are presented as the normalized activity of the indicated miR-transfected cells relative to the negative mimic control (miR-Ctr). These results represent the mean of at least three independent experiments ± standard error (SE), each carried out in triplicate. The significance levels were obtained by ANOVA: **, p<0.01; ***, p<0.001 compared with the control miRNA. (C) Combined miR-101/miR-494 synthetic mimics (100 nM miR-101 and 100 nM miR-494) or 200 nM miR-Ctr (^§^) were delivered into HEK293 cells together with pCFTR-3’UTR plasmid. Luciferase activity measured and data reported as in B. Statistical significance from ANOVA: ns, not significant; **, p<0.01; ***p<0.001 compared with the control miRNA; miR-101+miR494 versus miR-Ctr either at 100 nM or at 200 nM.

**Figure 3:**
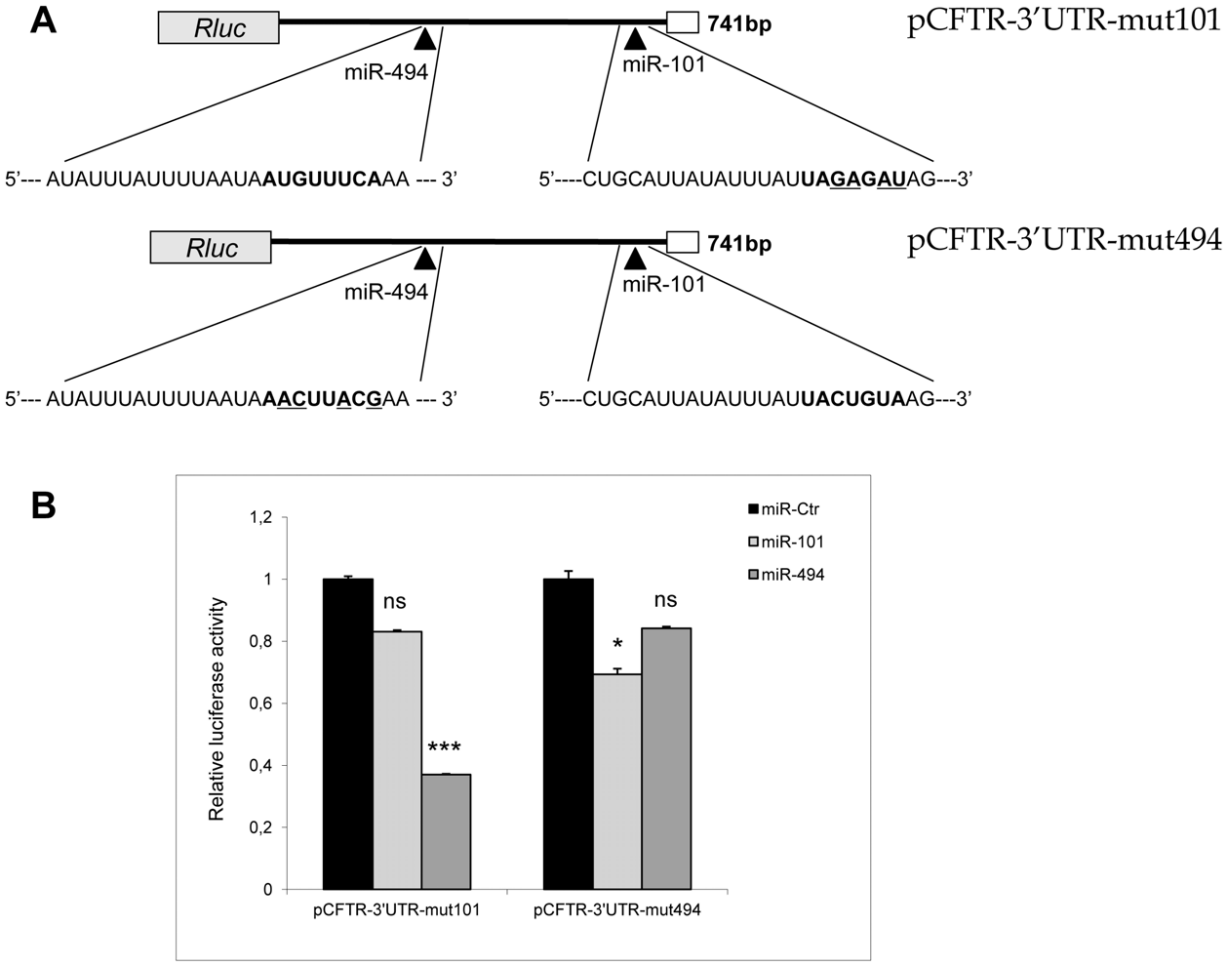
Specificity of miR-101 and miR-494 *CFTR* suppression by recognition of the seed sequence. (A) Schematic representation of the reporter constructs carrying *CFTR* 3’UTR mutated at miR-101 responsive element or mir-494 seed sequence. (B) Relative luciferase activity in HEK293 cells over-expressing the indicated miRNAs and transfected with the *CFTR* wild-type 3’UTR vector or its mutant derivative pCFTR-3’UTR-mut101 and pCFTR-3’UTR-mut494. Data are presented as the normalized activity of miR-transfected cells (miR-101 or miR-494) relative to cells transfected with miR-Ctr. All data are average values ± SE from at least three experiments (ANOVA, ns, not significant; *, p<0.05; ***, p<0.001 compared to the basal value with miR-Ctr).

To verify whether miR-101 and/or miR-494 were able to target *CFTR*, HEK293 cells were co-transfected either with a reporter construct containing 741 base pairs of the human *CFTR* 3’UTR downstream of the Renilla luciferase open reading frame (Figure 2A) or with a control Renilla plasmid together with either a synthetic microRNA mimics (miR-101 or miR-494) or the negative control microRNA (miR-Ctr). Both miR-101 and miR-494 significantly suppressed luciferase expression compared to control microRNA with a corresponding decrease of about 40% (p<0.01) and 60% (p<0.001) as shown in Figure 2B. miR-494 was also able to inhibit pCFTR-3’UTR in a dose-dependent manner and at low doses since the Renilla activity was significantly reduced of about 30% using a 10 nM miR-concentration (Figure S1). Notably, when miR-101 and miR-494 were co-overexpressed, a synergistic effect between miRNAs was observed, as highlighted by the strong reduction of the pCFTR-3’UTR reporter activity of approximately 80% (p<0.001). No significant decrease was evident when HEK293 cells were transfected with a double dose of microRNA negative control (Figure 2C). The MRE specificity in the *CFTR* 3’UTR was evaluated by site-directed mutagenesis of the nucleotides at positions 3, 4, 6, 7 of the miR-101 seed sequence (TACTGTA to TA**GA**G**AT**) and at position 2, 3, 6, 8 of the miR-494 target site (ATGTTTCA to A**AC**TT**A**C**G**) (Figure 3A). No repression was observed with pCFTR-3’UTR constructs in which the putative miRNA-binding sites for miR-101 or miR-494 were altered (Figure 3B). In particular, the activity of the reporter construct mutated at the specific miR-101 seed (pCFTR-3’UTR-mut101) was unaffected by the concomitant transfection of miR-101 and led to a significant decreased luciferase value only with miR-494 over-expression (p<0.001). At the same time, pCFTR-3’UTR-mut494 reporter activity was significantly reduced by miR-101 (p<0.05) while miR-494 transfection was nearly inactive. These data suggest that modifications of the specific miRNA binding-sites in the *CFTR* 3’UTR are able to reduce the inhibitory function of miR-101 and miR-494 (Figure 3B). Thus, miR-101 and miR-494 functionally interact with the *CFTR* 3’-UTR and suppress the corresponding protein product.

## Discussion

Since aberrant level and function of microRNAs have been recently observed in Cystic Fibrosis tissues [13]-[15], [18], the possibility they may act as phenotype modifiers was raised. Our data, together with the very recent publication by Gillen et al., [18] support a role of some microRNAs in the post-transcriptional regulation of the CFTR channel synthesis. The *CFTR* mRNA 3’-UTR, the putative site of miRNA annealing, is highly conserved in different species and contains several regulatory motifs important for the mRNA stability and translation control [19]. We identified a few miRNA-3’-UTR possible pairing with similar expression profiles and selected miR-101 and miR-494 for further analysis, since they showed the best possibility of positive interaction. Luciferase *in vitro* experiments confirmed that both miR-101 and miR-494 targeted and functionally suppressed *CFTR* construct mRNA translation with a moderate and strong action, respectively, and this activity was almost lost after mutation of the putative 3' UTR target-sites. The different strength of the luciferase repression could be due to the secondary structure MRE accessibility, the relative position of the specific target sites in the *CFTR* 3’-UTR as well as the different seed match types. Furthermore, miR-101 and miR-494 seem to act synergistically on CFTR-reporter inhibition with a more than additive effect on the post-translational control and this could have a physiological relevance in the complex disease phenotypes observed in CF. Interestingly, our results are corroborated by expression profiling experiments of different human miRNAs from CF patients airway epithelial samples in which both miR-101 and miR-494 looked up-regulated (Relative Quantification ≥1.5) compared to non-CF individuals confirming an inverse correlation with *CFTR* levels [13]. In particular, miR-494 levels were altered in four of the five CF samples showing an up to 6-fold increase respect to normal airway brushings. Up-regulation of miR-494 in asthmatic samples [20] and direct influence in cell cycle progression of primary murine bronchial epithelial cells exposed to carcinogens [21], [22] suggest that miR-494 altered levels have effects mainly in the lung compartment. As far as mir-101 concerns, recent evidences underline its role in the biosynthesis of pro-inflammatory cytokines [23] and its function in central nervous system [24] providing a possible explanation for both lung excessive inflammation and neural symptoms observed in CF patients [25]. Our study shows the possible relevance of miRNA pathways as genetic modifiers that may contribute to the varied CF phenotypes seen even among individuals carrying the same *CFTR* gene mutations. We speculate that microRNAs are largely responsible for how badly the illness affects patients since even minimal alterations in miR-101 and/or miR-494 levels could negatively influence the stability of the *CFTR* transcript which in turn might have an effect on the amount and maturation of *CFTR* protein and so on the degree of CF severity. This is well proved by the miRNA dose-dependent luciferase decreased activity and the concerted inhibitory interaction with *CFTR* 3′-UTR sequence showed in our *in vitro* results. Recent evidences also suggest that RNA binding proteins and microRNAs can regulate transcripts involved in a common pathway [26]. In particular, Spence [19] identified conserved regulatory motifs in the *CFTR* 3’-UTR that had a partial identity with 3’-UTR of *SEC24*, a gene coding for a protein implicated in the CFTR translocation out of the endoplasmic reticulum. Our preliminary *in silico* analysis found that both miR-101 and miR-494 may target *SEC24* 3’-UTR and two other genes, *TGFB1* and *MBL2*, which are known to modify the development and/or the severity of lung disease in CF [2], suggesting the existence of a coordinated network of gene expression control by microRNAs. Therefore, alteration of miR-101 and/or miR-494 levels in CF patients could influence the disease clinical expression with particular implications in CF lung function, such as increased susceptibility to infections, chronic airways inflammation and response to specific therapies. These miRNAs, as CF genetic modifiers, might act either by directly influence *CFTR* gene expression or/and other functionally related genes. Future investigations will be addressed to confirm this hypothesis such as the characterization of the entire *CFTR* 3’-UTR in a panel of CF subjects carrying the same mutations and different clinical phenotype looking for sequence variants in the miR-responsive elements that could dramatically alter *CFTR* regulation as well as mutations in the miR-101 and miR-494 genes which could explain their altered expression.

In conclusion, the discovery of miRNAs directly controlling *CFTR* regulation could have an enormous impact in the elucidation of CF pathology and might also contribute to clarify the biological causes of the phenotype/genotype discrepancies observed among CF patients. In addition, miR-based diagnostic and therapeutic applications represent an exciting possibility for this common mendelian disease. As for Duchenne muscular dystrophy [27], [28], quantification of selected miRNAs might be used as a sensitive biomarker tool of CF severity and functional suppression of *CFTR*-targeting miRNAs, such as miR-101 and/or miR-494, could prove a strategy to efficiently restore CFTR synthesis in patients carrying mutations leading to insufficient protein expression.

## Supporting Information

Figure S1 **Dose-dependent miR-inhibition of the** ***CFTR*** **luciferase reporter.** Levels of luciferase activity in HEK293 cells co-transfected with increasing doses of miR-101 or miR-494 together with the *CFTR* wild-type 3’-UTR vector. miR-494 was able to significantly inhibit the reporter activity in a dose-dependent manner and at low concentrations. All data are average values ± SE from three independent experiments, each carried out in triplicate. Statistical comparisons were performed by ANOVA (ns, not significant; *, p<0.05; **, p<0.01; ***, p<0.001 compared to the basal value).
(TIF)
