# Supplementary figures and images for: Synergistic Post-Transcriptional Regulation of the Cystic Fibrosis Transmembrane conductance Regulator (CFTR) by miR-101 and miR-494 Specific Binding
Source: PLoS One. 2011 Oct 20;6(10):e26601. doi: 10.1371/journal.pone.0026601 (PMC3197680; doi:10.1371/journal.pone.0026601)

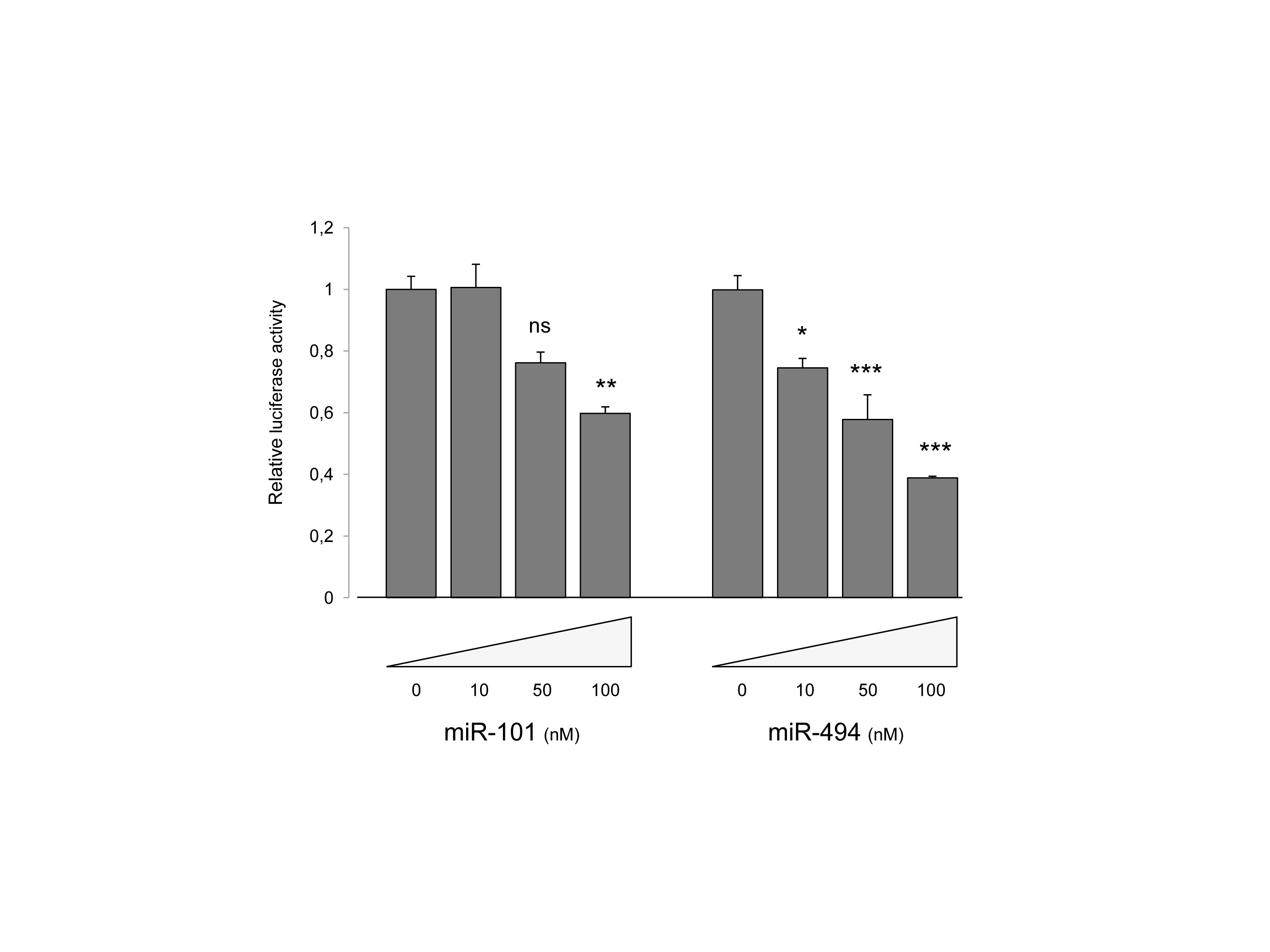

Supplement: Figure S1 — Dose-dependent miR-inhibition of the CFTR luciferase reporter. Levels of luciferase activity in HEK293 cells co-transfected with increasing doses of miR-101 or miR-494 together with the CFTR wild-type 3’-UTR vector. miR-494 was able to significantly inhibit the reporter activity in a dose-dependent manner and at low concentrations. All data are average values ± SE from three independent experiments, each carried out in triplicate. Statistical comparisons were performed by ANOVA (ns, not significant; *, p<0.05; **, p<0.01; ***, p<0.001 compared to the basal value). (TIF) [file pone.0026601.s001.tif]
